# Supplementary material for: Vortex conveyor belt for matter-wave coherent splitting and interferometry
Source: Sci Rep. 2019 Feb 4;9:1267. doi: 10.1038/s41598-019-38641-4 (PMC6362218; doi:10.1038/s41598-019-38641-4)
Supplement: Supplementary file 1 — Supplementary Information [file 41598_2019_38641_MOESM1_ESM.docx]

Supplementary Information

Vortex conveyor belt for matter-wave coherent
splitting and interferometry

Jixun Liu^1,2,*^, Xi Wang^2^, Jorge Mellado Muñoz^2^, Anna Kowalczyk^2^, and Giovanni Barontini^2^

^1^Institute of Optics and Electronics Technology, School of Instrumentation and Optoelectronic Engineering, Beihang University, Beijing, 100191, China

^2^Midlands Ultracold Atom Research Centre, School of Physics and Astronomy, University of Birmingham, Edgbaston, Birmingham, B15 2TT, United Kingdom

[*liujixun@buaa.edu.cn](mailto:*liujixun@buaa.edu.cn)


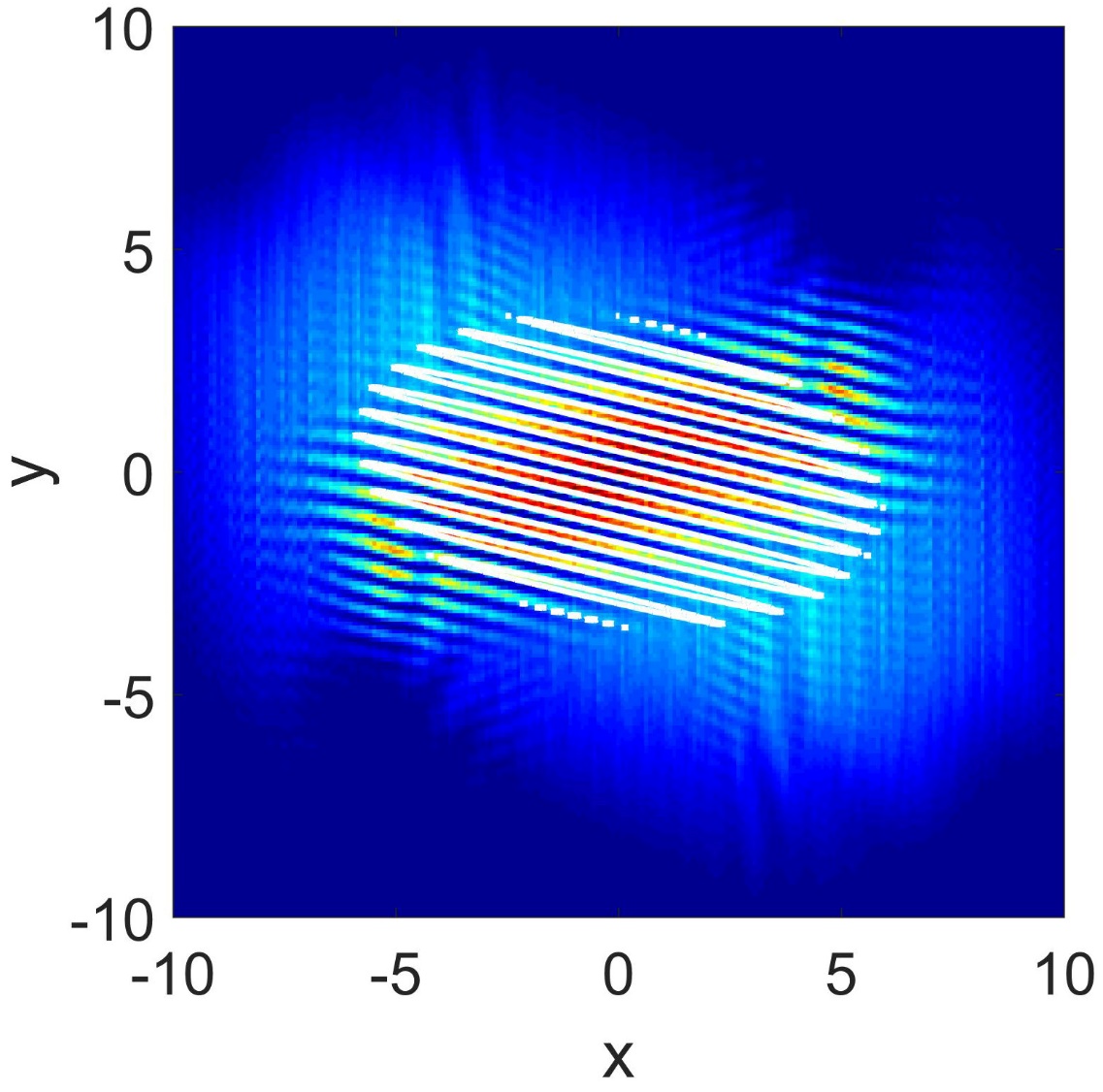


**Figure S1.** A comparison between the interference pattern and the fitting result. The column density profile chosen here showing the interference pattern is exactly the same with the one shown in Fig. 2 (f) in the main article. The white lines show the half maximum positions of the fitting result obtained with Eq. (6) in the main article.
